# Investigation of Wavenumber Domain Imaging Algorithm for Ground-Based Arc Array SAR

**DOI:** 10.3390/s17122950

**Published:** 2017-12-19

**Authors:** Zengshu Huang, Jinping Sun, Weixian Tan, Pingping Huang, Kuoye Han

**Affiliations:** 1 Electronics & Information Engineering, Beihang University, Beijing 100191, China; zengshu_huang@163.com; 2 College of Information Engineering, Inner Mongolia University of Technology, Hohhot 010051, China; wxtan@imut.edu.cn (W.T.); hwangpp@imut.edu.cn (P.H.); 3 Information Science Academy, China Electronics Technology Group Corporation, Beijing 100098, China; kuoyehan@hotmail.com

**Keywords:** arc antenna array, ground-based SAR, numerical solution, wavenumber domain, wide view angle imaging

## Abstract

Ground-based synthetic aperture radar (GB-SAR) has become an important technique for remote sensing deformation monitoring. However, most of the existing GB-SAR systems realize synthetic aperture by exploiting two closely spaced horn antennas to move along a linear rail. In order to obtain higher data acquisition efficiency and a wider view angle, we introduce arc antenna array technology into the GB-SAR system, which realizes a novel kind of system: ground-based arc array SAR (GB-AA-SAR). In this paper, we analyze arc observation geometry and derive analytic expressions of sampling criteria. Then, we propose a novel wavenumber domain imaging algorithm for GB-AA-SAR, which can achieve high image reconstruction precision through numerical solutions in the wavenumber domain. The proposed algorithm can be applied in wide azimuth view angle scenarios, and the problem of azimuth mismatch caused by distance approximation in arc geometric efficient omega-k imaging can be solved successfully. Finally, we analyze the two-dimensional (2D) spatial resolution of GB-AA-SAR, and verify the effectiveness of the proposed algorithm through numerical simulation experiments.

## 1. Introduction

Landslides, mudslides, and other deformation disasters seriously endanger people’s personal safety, as well as the safety of property. As a new type of technology for microwave remote sensing deformation measurement, ground-based synthetic aperture radar (GB-SAR) has been widely regarded for its extensive application prospects in deformation monitoring. Many research institutions have successfully developed GB-SAR systems, which also have been applied to deformation monitoring and disaster prevention [1,2,3,4,5,6,7]. The GB-SAR interferometry exploits non-contact microwave remote sensing measurement, which can achieve millimeter or even sub-millimeter level deformation monitoring accuracy [1,2]. The conventional GB-SAR system obtains high-range resolution by controlling antennas to transmit and receive wideband step frequency continue wave (SFCW) signals in the range direction, and the radar moves along a linear or arc mechanical rail to realize synthetic aperture. Then, the two-dimensional (2D) resolving of the observation area can be achieved. For catering to the development trends of miniaturization, lightness, and portability, the frequency modulation continuous wave (FMCW) signal has been applied into the design of the GB-SAR system. In order to further upgrade and optimize the existing GB-SAR system, this paper applies the arc antenna array technology to the GB-SAR system, which can obtain higher data acquisition efficiency and a wider view angle than conventional GB-SAR.

Arc antenna array technology is the technology that combines the arc observation geometry with an antenna array configuration. In [8], Klausing Helmut and Keydel Wolfgang firstly stated that a synthetic aperture can be obtained via the rotational movement, then subsequently proposed the corresponding imaging algorithms. Arc GB-SAR has attracted a great amount of interest in recent years, and some institutions have already developed their own prototype systems [9,10,11]. However, most of the current arc GB-SAR systems exploit the movement of a single pair of antennas rather than utilize an antenna array. The Joint Research Centre (JRC) has developed an array antenna system called MELISSA, but the observation geometry is equivalent to a liner array [12,13]. In this paper, an arc antenna array configuration is applied to the design of the GB-SAR system, which is referred to as ground-based arc array SAR (GB-AA-SAR). High-range resolution can be achieved for a FMCW signal with a large time-width product, whereas in the azimuth direction, the switched array antenna channels scan quickly over an arc aperture, and finally, high-speed and wide view angle data acquisition is realized.

This paper is organized as follows. In Section 2, the configuration of the GB-AA-SAR system and arc observation geometry are firstly introduced. Then, the corresponding arc array sampling criteria is derived, and the comparison between GB-AA-SAR and ground-based linear array SAR (GB-LA-SAR) is given. In Section 3, a novel wavenumber domain imaging algorithm for GB-AA-SAR is proposed, and the attainable spatial resolutions are analyzed. Finally, in Section 4, the GB-AA-SAR concept and the proposed imaging algorithm are validated by numerical simulations, and this paper is concluded in Section 5.

## 2. Ground-Based Arc Array SAR System

### 2.1. Arc Antenna Array and System Configuration

**Figure 1:**
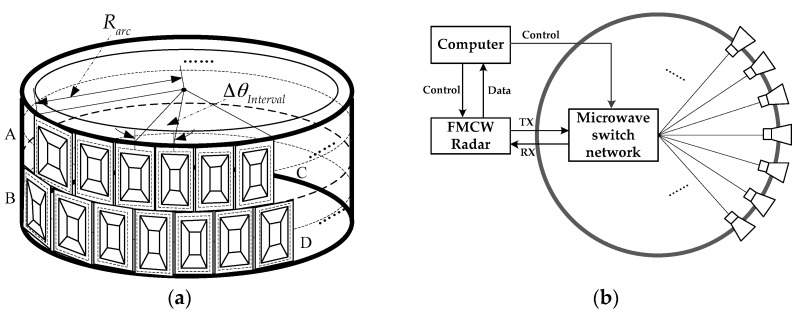
Ground-based arc array synthetic aperture radar (GB-AA-SAR) system: (**a**) Arc antenna array; (**b**) Configuration of GB-AA-SAR system.

Figure 1a shows the designed arc antenna array, where “AC” represents the transmitting antennas, and “BD” represents the receiving antennas. The transmitting and receiving antennas distribute uniformly along an arc, where the angle interval between adjacent antenna elements is fixed to be Δ*θ_Interval_*, and the radius of the arc is *R_arc_*. It is worth noting that the horn antenna element is adopted in this paper; on one hand, the horn antenna usually has a higher gain than that of other antenna forms, on the other hand, antennas such as microstrips cannot always support a wide bandwidth as much as the one used in this paper, which has reached up to 1 GHz. According to the equivalent phase center principle, a pair consisting of a transmitting antenna and a receiving antenna working in the quasi-stationary mode can be equivalent to a co-located virtual phase center. Thus, a uniform sampling of the arc array can be achieved. The transmitting and receiving antennas can be arranged in a staggered manner, which can realize single-transmitting and double-receiving data acquisition, and bring the benefits of improving data acquisition efficiency and the azimuth sampling rate [14]. As shown in Figure 1b, our GB-AA-SAR system is composed of three sub-blocks: an arc antenna array, a microwave switch network, and an FMCW transmitting/receiving (T/R) module. A microwave switch network can sequentially choose the antenna elements of arc array, and the FMCW T/R module also needs to transmit and receive the electromagnetic signal synchronously to finish the data acquisition. Compared with the mechanical movement of conventional GB-SAR systems, the electronic scanning mode of arc antenna array has outstanding advantages, such as for example, efficient data acquisition, a wide view angle, and a stable azimuth-sampling position in azimuth. Therefore, the GB-AA-SAR has great application potential in remote sensing deformation monitoring.

**Figure 2:**
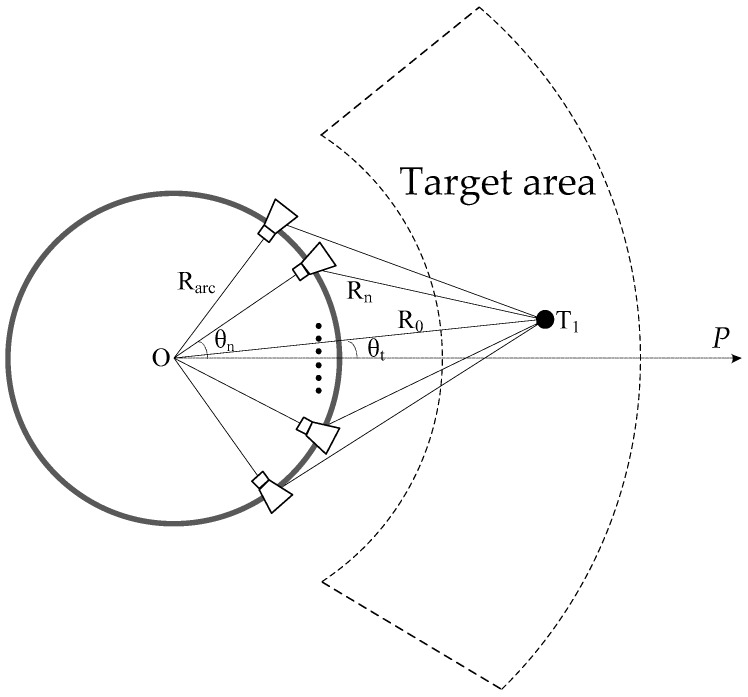
Observation geometric model of GB-AA-SAR.

GB-AA-SAR is usually applied in the deformation monitoring of slopes, dams, bridges, etc., which often lie in the far field of the antenna. The observation geometry of the GB-AA-SAR system is shown in Figure 2, where *O* is the center of the arc array, *θ_n_* is the angle between the nth antenna element and coordinate axis *OP*, *R*_0_ is the distance from target to center *O*, *R_n_* is the distance from target to the nth antenna element, and *θ_t_* is the angle between the target and coordinate axis *OP*. According to the geometric model, the distance from a point target located at (*R*_0_, *θ_t_*) to the nth antenna element can be expressed as:

(1) $$R_{n}=\sqrt{R_{0}^{2}+R_{\mathrm{arc}}^{2}-2R_{0}R_{\mathrm{arc}}\cos\left(\theta_{n}-\theta_{t}\right)}.$$

For the FMCW GB-AA-SAR system, the transmit signal is:

(2) $$S_{T}(t)=\mathrm{rect}\left(\frac{t}{T}\right)\exp\left\{j2\pi \left(f_{c}t+\frac{1}{2}Kt^{2}\right)\right\},$$

where $\mathrm{rect}\left(\frac{\cdot }{T}\right)$ represents the rectangular envelope with duration *T*, *t* is the fast time, *T* is the pulse duration, *f_c_* is the center frequency, *K* is the frequency modulation slope, and *B = KT* is the signal bandwidth. If the reflectivity of the point target is σ, the echo signal can be expressed as:

(3) $$S_{R}({t,R}_{n})=\sigma \cdot \mathrm{rect}\left(\frac{t-\frac{2R_{n}}{c}}{T}\right)\exp\left\{j2\pi \left(f_{c}\left(t-\frac{2R_{n}}{c}\right)+\frac{1}{2}K{\left(t-\frac{2R_{n}}{c}\right)}^{2}\right)\right\},$$

where *c* is the propagation speed of the electromagnetic wave. Then, the intermediate frequency (IF) signal after dechirp can be expressed as:

(4) $$S_{\mathrm{IF}}(t,\;\theta_{n})=\sigma \cdot w_{r}\left(t-\frac{2R_{n}}{c}\right)\cdot w_{a}\left(\theta_{n}-\theta_{t}\right)\exp\left\{-j2\pi \left(\left(f_{c}+Kt\right)\frac{2R_{n}}{c}-\frac{1}{2}K{\left(\frac{2R_{n}}{c}\right)}^{2}\right)\right\},$$

where $w_{a}\left(\theta \right)=\mathrm{rect}\left(\frac{\theta }{\theta_{a}}\right)$ is the antenna beam pattern [11], and $-\frac{1}{2}K{\left(\frac{2R_{n}}{c}\right)}^{2}$ is the residual video phase (RVP) [14].

For the FMCW signal, the RVP in the IF signal is related to frequency modulation slope K and distance $R_{n}$, so it needs to be compensated in the spatial domain. The signal after Fourier transform is described as:

(5) $$S_{\mathrm{IFFT}}(t,\;\theta_{n})=\sigma \cdot p_{r}\left(t-\frac{2R_{n}}{c}\right)\cdot w_{a}\left(\theta_{n}-\theta_{t}\right)\exp\left\{-j2\pi \left(f_{c}\frac{2R_{n}}{c}-\frac{1}{2}K{\left(\frac{2R_{n}}{c}\right)}^{2}\right)\right\},$$

where $p_{r}\left(t\right)$ is the point spread function in the range direction.

Then, the RVP can be compensated by multiplying it with the following spatial-varying compensation terms [14]:

(6) $$H=\exp\left\{-j\pi K{\left(\frac{2R_{n}}{c}\right)}^{2}\right\}.$$

The spatial domain signal after compensation is:

(7) $$S_{\mathrm{RVP}}(t,\;\theta_{n})=\sigma \cdot p_{r}\left(t-\frac{2R_{n}}{c}\right)\cdot w_{a}\left(\theta_{n}-\theta_{t}\right)\exp\left\{-j\frac{4\pi f_{c}}{c}R_{n}\right\}.$$

### 2.2. Sampling Criteria of Ground-Based Arc Array SAR

In order to avoid aliasing during the imaging, the GB-AA-SAR system must obey the Nyquist sampling criterion. For fast-time domain sampling, the frequency domain support band of the dechirped echo signal is determined by:

(8) $$\Omega_{k}=\left[k_{r\min},\;k_{r\max}\right],$$

where $k_{r}=\frac{2\pi f}{c}$ is the range wavenumber, $k_{\mathrm{rmin}}=\min\left\{k_{r}\right\}$ is the minimum value of the range wavenumber, $k_{\mathrm{rmax}}=\max\left\{k_{r}\right\}$ is the maximum value of the range wavenumber. In the range direction, when Fourier analysis is performed, the range sampling rate needs to satisfy the following condition [14]:

(9) $$\{\begin{array}{l} \Delta R\leq \frac{2\pi }{2\left(k_{r\max}-k_{r\min}\right)}\\ F_{s}=\frac{c}{2\Delta R}\geq \frac{c\left(k_{r\max}-k_{r\min}\right)}{2\pi } \end{array},$$

where ΔR is the range resolution cell.

For the angle sampling interval in the azimuth direction, the instantaneous angular frequency can be expressed as:

(10) $$k_{\theta }^{\prime }=\frac{\partial \left[\arg\left(S_{\mathrm{RVP}}\right)\right]}{\partial \theta_{n}}=2k_{r}\frac{R_{arc}R_{0}\sin(\theta_{n}-\theta_{t})}{\sqrt{R_{arc}{}^{2}+R_{0}{}^{2}-2R_{arc}R_{0}\cos(\theta_{n}-\theta_{t})}},\;\theta_{n}\in \left[\theta_{t}-\theta_{d},\;\theta_{t}+\theta_{d}\right],$$

where $\theta_{d}$ is the maximum angle difference between all of the antenna elements and the zero Doppler center of the observed target. Then, the angle-sampling interval in azimuth can be derived as follows:

(11) $$\Delta \theta \leq \frac{2\pi }{k_{\theta \max}^{\prime }-k_{\theta \min}^{\prime }}=\frac{c\sqrt{R_{arc}{}^{2}+R_{0}{}^{2}-2R_{arc}R_{0}\cos(\theta_{d})}}{4R_{arc}R_{0}\sin(\theta_{d})f},$$

where $k_{\theta \max}^{\prime }$ is the maximum value of $k_{\theta }^{\prime }$, and $k_{\theta \min}^{\prime }$ is the minimum value of $k_{\theta }^{\prime }$. The azimuth angle-sampling interval in Equation (11) depends on the system frequency, the target distance, and the beamwidth of the antenna element. However, the arrangement of the arc antenna array requires a specific sampling interval, so we will combine antenna beamwidth with arc observation geometry for further analysis.

**Figure 3:**
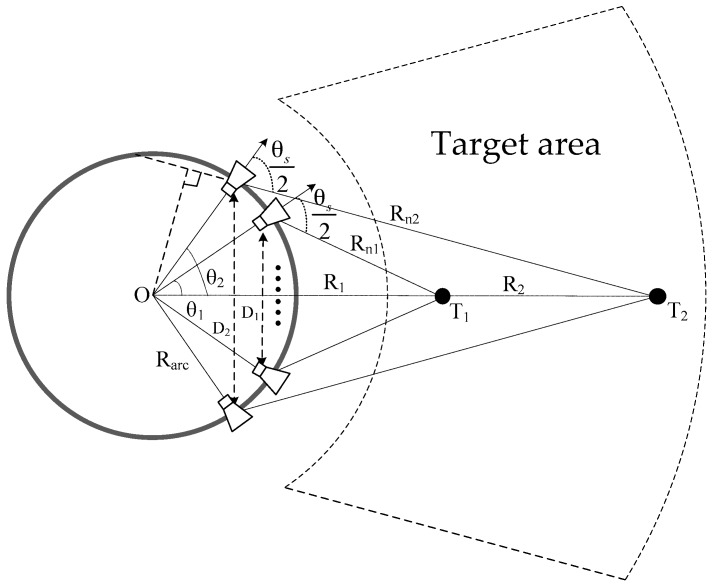
Geometric model for azimuth sampling analysis.

Figure 3 shows the geometric model of GB-AA-SAR for targets at different distances, which takes into account the effect of antenna beamwidth on the observed target. The antenna beamwidth is $\theta_{s}$. The farther the target is, the more antenna elements will illuminate the target, and vice versa. Firstly, taking the far target T2 as an example, according to Equation (11), the angle-sampling interval can be expressed as follows:

(12) $$\Delta \theta_{2}\leq \frac{c\sqrt{R_{arc}{}^{2}+R_{2}{}^{2}-2R_{arc}R_{2}\cos(\theta_{2})}}{4R_{arc}R_{2}\sin(\theta_{2})f}$$

The following relationship can be obtained, according to the observation geometry in Figure 3.

(13) $$\{\begin{array}{l} R_{n2}=\sqrt{R_{arc}{}^{2}+R_{2}{}^{2}-2R_{arc}R_{2}\cos(\theta_{2})}\\ D_{2}=2R_{arc}\sin\left(\theta_{2}\right)\\ \frac{D_{2}}{2R_{n2}}=\sin\left(\frac{\theta_{s}}{2}-\theta_{2}\right)\\ R_{2}\sin\left(\frac{\theta_{s}}{2}-\theta_{2}\right)=R_{arc}\sin\left(\frac{\theta_{s}}{2}\right) \end{array}$$

Substituting Equation (13) into Equation (12), the angle-sampling interval is available:

(14) $$\Delta \theta_{2}\leq \frac{c}{4R_{arc}\sin(\frac{\theta_{s}}{2})f}$$

The same conclusion can be obtained for the near target T1, so the azimuth angle-sampling interval of the arc array is independent of the target distance. Further to analysis, taking the maximum frequency $f_{\max}$ into Equation (14), the sampling interval in the azimuth direction can be expressed as:

(15) $$\Delta \theta \leq \frac{\lambda_{\min}}{4R_{arc}\sin(\frac{\theta_{s}}{2})},$$

where $\lambda_{\min}{=c/f}_{\max}$ is the minimum wavelength. It can be seen that the azimuth angle-sampling interval of the arc array is only related to shortest wavelength, arc radius, and antenna beamwidth, which are known in the system design process. Therefore, the arrangement of the antenna elements is available.

### 2.3. Comparison with GB-LA-SAR

In order to illustrate the advantages of GB-AA-SAR compared with GB-LA-SAR, a comparison of both configurations are demonstrated in Table 1. Single-transmitting and double-receiving data acquisition are performed in both configurations. It can be seen when the rest of the parameters are given and similar in GB-AA-SAR and GB-LA-SAR, the view angle range of GB-AA-SAR is wider than that of GB-LA-SAR. Since the view angle range of GB-LA-SAR is limited by the antenna beamwidth, the view angle range of GB-AA-SAR is dependent on the length of the arc, which can be increased easily, even to the length of the circle. So, the GB-AA-SAR has the capability to achieve a wide view angle or even 360° deformation monitoring.

## 3. Ground-Based Arc Array SAR Wavenumber Domain Imaging Algorithm

### 3.1. Wavenumber Domain Imaging Algorithm for GB-AA-SAR

The wavenumber domain imaging is performed in a two-dimensional frequency domain. The spatial signal can be transformed into the frequency domain by fast Fourier transform (FFT), and the typical imaging algorithm is the omega-k algorithm [15,16]. However, for the arc observation geometry, the data acquisition of the SAR signal is realized in polar coordinate format. So, the two-dimensional FFT is not applicable, and the conventional omega-k imaging cannot be directly applied to arc observation geometry. Of course, the spatial interpolation can realize the transformation from polar coordinates to Cartesian coordinates. However, the interpolation requires some computation. More importantly, a large number of zero-padding operations in azimuth are required, which will bring a great burden on the memory and calculation [15]. Therefore, this scheme is not suitable for the GB-AA-SAR imaging with a wide view angle. Dallinger has proposed an efficient omega-k algorithm in polar coordinates [16]. However, due to the Taylor approximation in the efficient omega-k algorithm, the mismatch problem in azimuth is brought about, which is unacceptable for GB-AA-SAR wide view angle imaging. The parameter $k_{y}$ in Dallinger’s algorithm is related to the target distance. For airborne and spaceborne SAR imaging, since the observation scene is much smaller than the distance from the observation scene to radar, the reference distance can take the center of the observation scene. However, the approximation is not precise in a ground-based platform, so the algorithm is not suitable for GB-AA-SAR imaging.

For the signal $S_{\mathrm{RVP}}(t,\theta_{n})$ shown in Equation (7), range Fourier transform is applied, and the frequency domain signal can be expressed as follows:

(16) $$S_{k_{r},\theta_{n}}=\sigma \cdot w_{r}\left(\frac{{f-f}_{c}}{B}\right)\cdot w_{a}\left(\theta_{n}-\theta_{t}\right)\exp\left\{-{j2k}_{r}R_{n}\right\},$$

where $f=f_{c}+Kt$ is the signal frequency. After Fourier transform in azimuth, the signal in the two-dimensional spatial frequency domain could be described as follows:

(17) $$\begin{array}{ll} S_{k_{r},k_{\theta }} & =\int_{\theta_{n}}S_{k_{r},\theta_{n}}\cdot \exp\left(-j2\pi \frac{f_{\eta }}{w_{s}}\theta_{n}\right)d\theta_{n}\\ & =\int_{\theta_{n}}\sigma \cdot w_{r}\left(\frac{{f-f}_{c}}{B}\right)\cdot w_{a}\left(\theta_{n}-\theta_{t}\right)\exp\left\{-{j2k}_{r}R_{n}\right\}\cdot \exp\left(-jk_{\theta }\theta_{n}\right)d\theta_{n}, \end{array}$$

where $k_{\theta }=\frac{2\pi f_{\eta }}{w_{s}}$ is the spatial angular frequency in azimuth direction, the phase in Equation (17) is:

(18) $$\begin{array}{ll} \Phi \left(\theta_{n}\right) & =-2k_{r}R_{n}-k_{\theta }\theta_{n}\\ & =-2k_{r}\sqrt{R_{0}^{2}+R_{\mathrm{arc}}^{2}-2R_{0}R_{\mathrm{arc}}\cos\left(\theta_{n}-\theta_{t}\right)}-k_{\theta }\theta_{n}. \end{array}$$

In order to convert the spatial variant properties into spatial invariant properties, and realize the efficient omega-k imaging in polar coordinates for arc observation geometry, it is necessary to rewrite Equation (18) as follows:

(19) $$\Phi \left(\theta_{n}^{\mathrm{pos}}\right)=-{2k}_{r}\sqrt{R_{0}^{2}+R_{\mathrm{arc}}^{2}-2R_{0}R_{\mathrm{arc}}\cos\left(\theta_{n}-\theta_{c}\right)}-k_{\theta }\theta_{n}=P\left({k,k}_{\theta }\right)Q\left(R_{0}\right)+H\left(k_{\theta }\right)G(\theta_{c}),$$

where $P\left({k,k}_{\theta }\right)$ represents a function of ${k,\;k}_{\theta }$, $Q\left(R_{0}\right)$ represents a function of $R_{0}$, $H\left(k_{\theta }\right)$ represents a function of $k_{\theta }$, and $G(\theta_{c})$ represents a function of $\theta_{c}$. Therefore, the stationary phase point $\theta_{n}^{\mathrm{pos}}$ in Equation (19) could be solved through the Taylor expansion approximation and principle of stationary phase (POSP):

(20) $$\theta_{n}^{\mathrm{pos}}=\theta_{c}-\frac{\alpha \cdot k_{\theta }/\beta }{\sqrt{4k_{r}-k_{\theta }{}^{2}/\beta }},$$

where $\alpha =R_{0}-R_{\mathrm{arc}}$ and $\beta =R_{0}R_{\mathrm{arc}}$, taking Equation (20) into Equation (17), the two-dimensional spectrum in polar coordinates can be rewritten as follows:

(21) $$S_{k_{r},k_{\theta }}=\sigma \cdot w_{r}\left(k_{r}\right)\cdot w_{a}\left(k_{\theta }\right)\cdot \exp\left(-j\sqrt{4k_{r}^{2}{-k}_{\theta }^{2}/\beta }\cdot \alpha -jk_{\theta }\theta_{c}\right).$$

Then, a nonlinear mapping referred to as the STOLT interpolation [15] is performed on the two-dimensional spectrum, where the mapping relationship is:

(22) $$\{\begin{array}{l} k_{y}=\sqrt{4k_{r}^{2}-k_{\theta }^{2}/\beta }\\ \;k_{x}=k_{\theta } \end{array}.$$

Finally, the efficient omega-k imaging is finished by two-dimensional inverse fast Fourier transform (IFFT).

Due to the efficient omega-k algorithm not being suitable for GB-AA-SAR, this paper presents a novel wavenumber domain imaging algorithm for GB-AA-SAR. The focus process in the range direction is realized by numerical solution in the wavenumber domain, and then, azimuth FFT without zero padding is applied to obtain the accurate SAR image, specifically:

Firstly, let $v=\theta_{n}-\theta_{t}$, then Equation (17) can be rewritten as follows:

(23) $$\begin{array}{ll} S_{k_{r},k_{\theta }} & =\int_{\theta_{n}}\sigma \cdot w_{r}\left(\frac{f-f_{c}}{B}\right)\cdot w_{a}\left(v\right)\exp\left\{-{j2k}_{r}R_{n}\right\}\cdot \exp\left(-jk_{\theta }\left(v+\theta_{t}\right)\right)\mathrm{dv}\\ & =\int_{\theta_{n}}\sigma \cdot w_{r}\left(\frac{f-f_{c}}{B}\right)\cdot w_{a}\left(v\right)\exp\left\{-{j2k}_{r}\sqrt{R_{0}^{2}+R_{\mathrm{arc}}^{2}-2R_{0}R_{\mathrm{arc}}\cos\left(v\right)}\right\}\cdot \exp\left(-jk_{\theta }v\right)\mathrm{dv}\cdot \exp\left(-jk_{\theta }\theta_{t}\right). \end{array}$$

The phase in Equation (23) is:

(24) $$\Phi \left(v\right){=-2k}_{r}\sqrt{R_{0}^{2}+R_{\mathrm{arc}}^{2}-2R_{0}R_{\mathrm{arc}}\cos\left(v\right)}{-k}_{\theta }{v-k}_{\theta }\theta_{t}.$$

Then the stationary phase can be obtained by solving Equation (24):

(25) $$\frac{\partial \Phi \left(v\right)}{\partial v}=\frac{-{2k}_{r}R_{0}R_{\mathrm{arc}}\sin\left(v_{\mathrm{pos}}\right)}{\sqrt{R_{0}^{2}+R_{\mathrm{arc}}^{2}-2R_{0}R_{\mathrm{arc}}\cos\left(v_{\mathrm{pos}}\right)}}-k_{\theta }=0.$$

The result is derived as follows:

(26) $$\{\begin{array}{l} \cos\left(v_{\mathrm{pos}}\right)=\frac{k_{\theta }^{2}}{4\beta k_{r}}+\sqrt{\frac{k_{\theta }^{4}}{16k_{r}^{4}\beta^{2}}-\frac{k_{\theta }^{2}\alpha_{0}}{4k_{r}^{2}\beta^{2}}+1}\\ v_{\mathrm{pos}}=\arccos\left(\frac{k_{\theta }^{2}}{4\beta k_{r}}+\sqrt{\frac{k_{\theta }^{4}}{16k_{r}^{4}\beta^{2}}-\frac{k_{\theta }^{2}\alpha_{0}}{4k_{r}^{2}\beta^{2}}+1}\right) \end{array},$$

where $\alpha_{0}=R_{0}{}^{2}+R_{\mathrm{arc}}{}^{2}$, taking the results in Equation (26) into Equation (23), the result could be computed as follows:

(27) $$S_{k_{r},k_{\theta }}=\sigma \cdot w_{r}\left(\frac{f-f_{c}}{B}\right)\cdot w_{a}\left(v_{\mathrm{pos}}\right)\exp\left\{-j\left({2k}_{r}\sqrt{R_{0}^{2}+R_{\mathrm{arc}}^{2}-2R_{0}R_{\mathrm{arc}}\cos\left(v_{\mathrm{pos}}\right)}+k_{\theta }v_{\mathrm{pos}}\right)\right\}\cdot \exp\left(-jk_{\theta }\theta_{t}\right).$$

For the target in distance $R_{i}$, let the compensation phase be:

(28) $$\Psi_{i}={2k}_{r}\sqrt{R_{i}^{2}+R_{\mathrm{arc}}^{2}-2R_{i}R_{\mathrm{arc}}\cos\left(v_{\mathrm{pos}}^{i}\right)}+k_{\theta }v_{\mathrm{pos}}^{i}.$$

Then, the range accumulation is applied in the wavenumber domain:

(29) $$S_{R_{i},k_{\theta }}=\int_{\theta_{n}}S_{k_{r},\theta_{n}}\cdot \exp(j\Psi_{i})dk_{r}=\sigma \cdot p_{r}\left(t-\frac{2R_{i}}{c}\right)\cdot \exp\left(-jk_{c}R_{i}\right)\cdot w_{a}\left(\theta_{t}\right)\cdot \exp\left(-jk_{\theta }\theta_{t}\right).$$

For the observation scene with *N_r_* sampling points, the range profile can be obtained by performing the operation to all of the sampling distances in the observation scene, and then the accurate SAR image is obtained through FFT operation in the azimuth direction.

(30) $$S_{R,\theta }={\mathrm{FFT}}_{a}\left[S_{R_{i},k_{\theta }}\right]=\sigma \cdot p_{r}\left(t-\frac{2R_{0}}{c}\right)\cdot p_{a}\left(\theta -\theta_{t}\right)\cdot \exp\left(-jk_{c}R_{0}\right).$$

**Figure 4:**
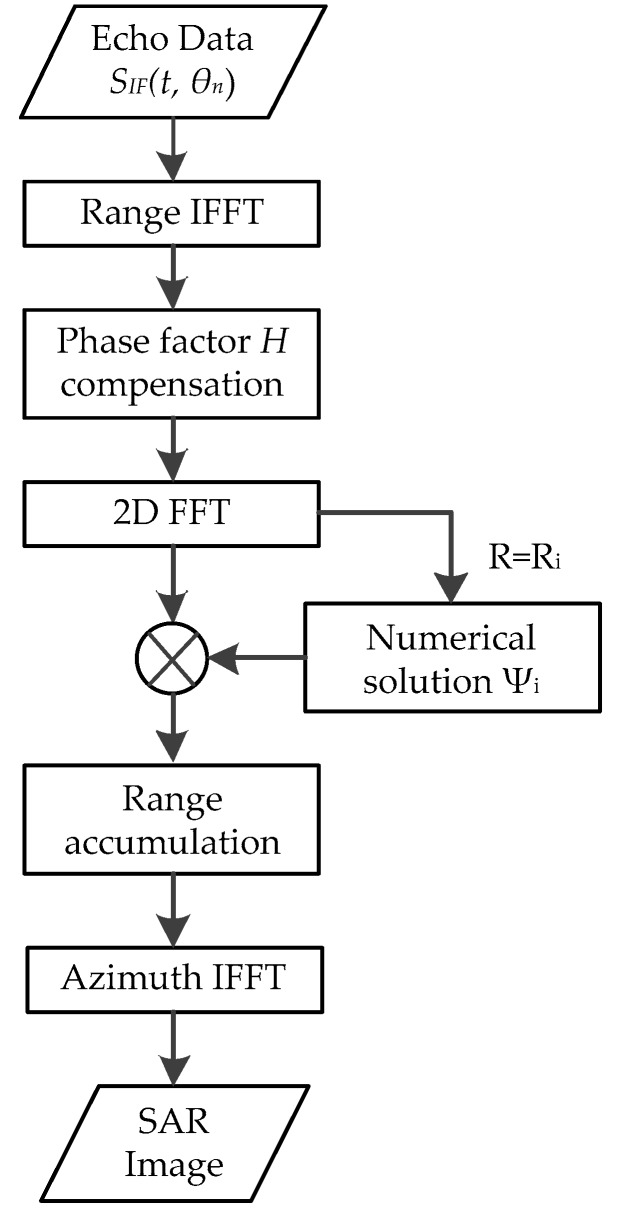
Wavenumber domain imaging processing flow chart.

In summary, the wavenumber domain imaging procedure of GB-AA-SAR is illustrated in Figure 4, which can be split into the following steps:

Step 1 Perform an IFFT on the measured echo data *S_IF_*(*t*, *θ_n_*) with respect to the fast time *t* in the range direction.
Step 2 Compensate the RVP using phase factor *H*, and obtain the result *S_RVP_*(*t*, *θ_n_*).
Step 3 Perform the 2D FFT of the *S_RVP_*(*t*, *θ_n_*) with respect to *t* and *θ_n_* to obtain the 2D frequency domain signal *S_k__r__, k__θ_*.
Step 4 For *R* = *R_i_*, realize a numerical solution in the wavenumber domain to acquire the stationary phase Ѱ*_i_*.
Step 5 Compensate the phase Ѱ*_i_*; then, perform range accumulation in order to accomplish the focus procedure at *R* = *R_i_*.
Step 6 Repeat Steps 4 to 5 for all of the available *R_i_*, which can be rapidly implemented by parallel processing, then perform azimuth IFFT to the accumulated result *S_R, k__θ_* to accomplish the focus procedure in the azimuth direction.
Step 7 Finally, display the SAR image.

### 3.2. Range Resolution and Azimuth Angular Resolution

For GB-AA-SAR, the point-spread function (PSF) of a point target is:

(31) $$\mathrm{PSF}\left(R,\;\theta \right)\sim \sin c\left\{\frac{B_{\mathrm{kr}}}{2\pi }R_{n}\right\}\sin c\left\{\frac{B_{k\theta }}{2\pi }\theta_{n}\right\},$$

where sinc(x)=sin(πx)/πx, $B_{\mathrm{kr}}$ is the wavenumber bandwidth in the range direction, and $B_{k\theta }$ is the wavenumber bandwidth in the azimuth direction. The resolution is usually defined as −3 dB bandwidth of the PSF main lobe. Then, according to the discussion in Section 2.2, the range resolution $\rho_{r}$ and the angular resolution $\rho_{\theta }$ can be expressed as follows:

(32) $$\{\begin{array}{l} \rho_{r}=\frac{0.886\times 2\pi }{2\left(k_{r\max}-k_{r\min}\right)}\;=\frac{0.886c}{2B}\\ \rho_{\theta }=\frac{0.886\times 2\pi }{2\left(k_{\theta \max}-k_{\theta \min}\right)}=\frac{0.886\lambda_{c}}{4R_{arc}\sin(\frac{\theta_{s}}{2})} \end{array}.$$

**Figure 5:**
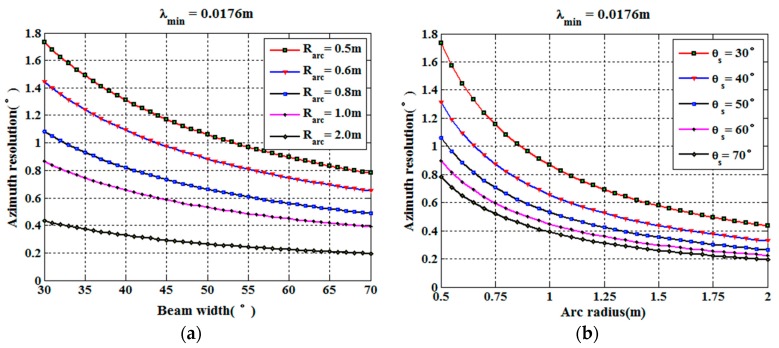
Azimuth angle resolution of GB-AA-SAR: (**a**) Azimuth angle resolution for different arc radii; (**b**) Azimuth angle resolution for different antenna beamwidths.

It can be seen that, if the system parameters are given, the azimuth angle resolution of GB-AA-SAR is a constant, which is independent of the target distance. Figure 5 shows the azimuth angle resolution curve of GB-AA-SAR for different antenna beamwidths and arc radii.

## 4. Numerical Simulation Experiments

In this section, numerical simulation experiments for GB-AA-SAR are performed. Firstly, point target imaging simulation is performed to verify the effectiveness of the proposed algorithm through comparing it with the back projection algorithm (BP) and efficient omega-k algorithm. Then, multi-point target imaging is carried out with different distances and azimuth angles, and the 2D resolution of GB-AA-SAR is analyzed. The spatial invariant property of the azimuth angle resolution and the correctness of its theoretical value are verified. Simulation parameters are shown in Table 2.

**Figure 6:**
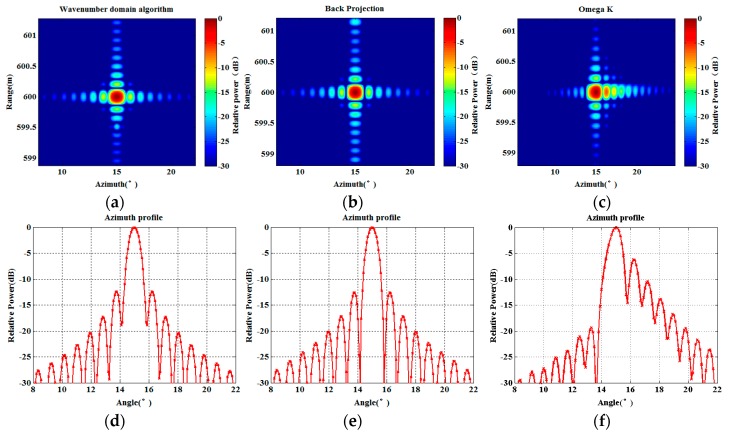
Two-dimensional (2D) imaging results of a point target and their respective azimuth profiles with different algorithms: (**a**,**d**) the proposed algorithm; (**b**,**e**) back projection algorithm; (**c**,**f**) omega-k algorithm.

The point target imaging simulation using the proposed wavenumber imaging algorithm is carried out. The imaging results are compared with the results of the BP algorithm and the efficient omega-k algorithm in order to verify the imaging performance of the proposed algorithm. Figure 6 shows the imaging results of the three algorithms. Figure 6a is the point target imaging result using the proposed wavenumber domain imaging algorithm, Figure 6b is the point target imaging result of the BP algorithm, and Figure 6c gives the result of the efficient omega-k algorithm. Correspondingly, Figure 6d–f show the azimuth profile of the three imaging algorithms. It can be seen that all of the algorithms can finish the point target focus. In order to quantitatively compare the imaging performance of the algorithms, we measure the image quality parameters: impulse response width (IRW), peak sidelobe ratio (PSLR), and integrated sidelobe ratio (ISLR). IRW is the main lobe width of impulse response value above −3 dB, which is also known as resolution [17].

All of the imaging processes are performed on the same computer with the same imaging range, which is [570 m,630 m]. The analysis results are shown in Table 3. It can be seen that the performance of the proposed algorithm is similar to the BP algorithm, but the proposed algorithm could be more efficient than the BP imaging algorithm. The proposed algorithm is inefficient compared with the omega-k algorithm, but can solve the problem of azimuth mismatch.

In Section 3.2, we analyzed the 2D resolution of the GB-AA-SAR system, and derived the analytic expression of the range resolution and the azimuth resolution. The conclusion showed that the azimuth angle resolution is independent of the target distance and only depends on the wavelength, the radius of the arc array, and the antenna beamwidth. So, the spatial resolution of GB-AA-SAR in polar coordinates has spatially invariant properties. For further study, an analysis of the targets with different observation distances and different azimuth angles should be performed, and two-dimensional IRW given to verify the above conclusions.

**Figure 7:**
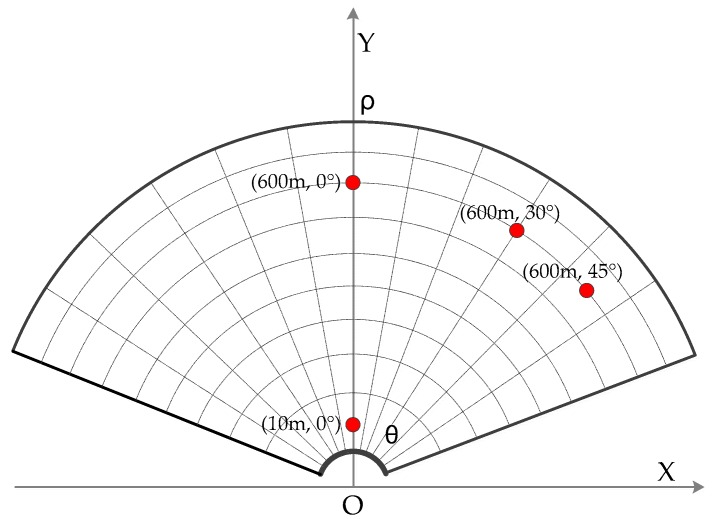
Distribution of the point targets.

**Figure 8:**
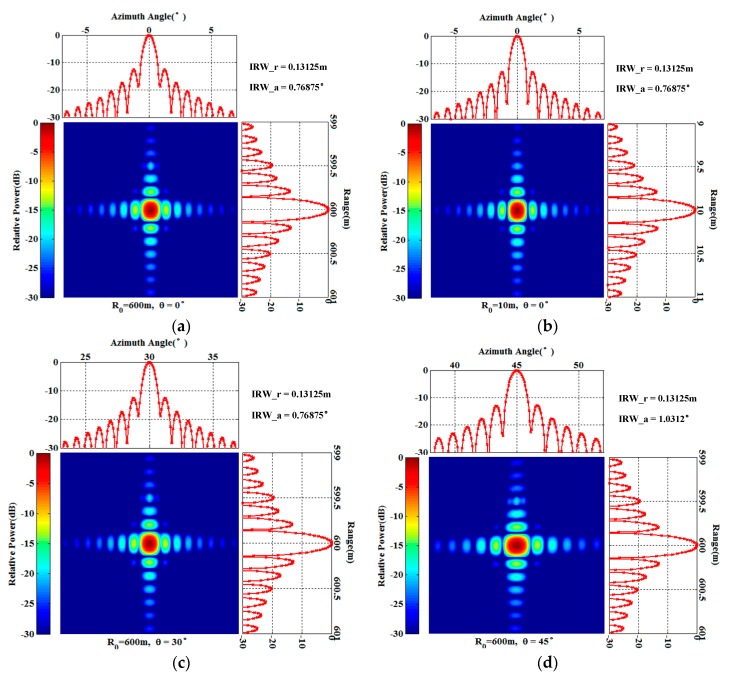
Imaging results of the targets with different distances and different azimuth angles. (**a**) Point targets at (600 m, 0°); (**b**) Point targets at (10 m, 0°); (**c**) Point targets at (600 m, 30°); (**d**) Point targets at (600 m, 45°).

In order to verify the 2D resolution sufficiently, point targets at (600 m, 0°), (10 m, 0°), (600 m, 30°), and (600 m, 45°) are analyzed respectively. The distribution of the targets is shown in Figure 7. As shown in Figure 8a,b, it can be seen that the resolutions are the same for the targets with different distances. Figure 8a,c shows the focus results of the targets with the same observation distance, but different azimuth angles. The resolutions are consistent with the conclusion and the theoretical values previously analyzed. However, in Figure 8d, when the azimuth angle of the target expanded to 45°, the azimuth resolution decreased. The reason is that the angle range of the arc array is designed as [−60°, 60°], when the target is at 45° in azimuth, there are not enough array antennas to illuminate the target [15,18]. Correspondingly, the azimuth resolution decreases with the reducing of the azimuth synthesis aperture, which is not contradictory to the conclusion that the azimuth resolution has spatially invariant properties. This phenomenon also occurs in the linear array case; thus, it is not a new phenomenon. In practice, if it is necessary to carry out regional observation with a super-wide view angle, and the cost is allowed, the optimal solution is to increase the arc length and the number of antenna elements. The sub-optimal solution is to realize data acquisition by the mechanical rotating of the arc array, which is low-cost but inefficient. Both solutions can realize high-resolution and super-wide view angle imaging processing.

## 5. Conclusions

In order to obtain efficient data acquisition and wide view angle imaging, the arc antenna array technology is applied into the GB-SAR system in this paper, which realizes a novel system: GB-AA-SAR. Then, we derived analytic expressions of sampling criteria, and proposed a novel wavenumber domain imaging algorithm suitable for GB-AA-SAR. Through comparing with the BP algorithm and efficient omega-k algorithm, it can be concluded that the proposed algorithm has the same accuracy as the BP algorithm, but the efficiency is higher than the BP algorithm. Further, the proposed imaging algorithm is inefficient compared with the omega-k algorithm, but can solve the problem of azimuth mismatch. Finally, we analyzed the resolutions of GB-AA-SAR and carried out multi-point targets numerical simulation, which not only verified the spatially invariant properties and the theoretical value of the resolutions, but also verified that the proposed imaging algorithm can finish high-precision imaging processing for wide azimuth view angle scenarios.

## Figures and Tables

**Table 1:** Comparison of GB-AA-SAR and ground-based linear array synthetic aperture radar (GB-LA-SAR).

Symbol	Parameters	GB-AA-SAR	GB-LA-SAR
*R_arc_*	Arc radius	0.6 m	— —
*θ_s_*	Antenna beamwidth	60°	60°
*L_a_*	Length	1.257 m	1.257 m
*N_a_*	Number of antennas (T/R)	71	71
*∆θ*	Sampling interval	0.843°	0.009 m
*θ_arc_*	View angle range	[−60°, 60°]	[−30°, 30°]

**Table 2:** System simulation parameters.

Symbol	Parameters	Value
*f_c_*	Center frequency	16.5 GHz
*B_r_*	Bandwidth	1 GHz
*R_arc_*	Arc radius	0.6 m
*K*	Frequency modulation rate	10^4^ GHz/s
$T_{r}$	Frequency modulation time	0.1 ms
*θ_s_*	Antenna beamwidth	60°
*L_a_*	Arc length	1.257 m
*θ_arc_*	Arc angle range	[−60°, 60°]
*N_a_*	Number of antennas (T/R)	71
*∆θ*	Angle sampling interval	0.843°

**Table 3:** Experimental analysis results. IRW: impulse response width; PLSR: peak sidelobe ratio; ISLR: integrated sidelobe ratio.

Measure Parameters		Our Method	*BP*	Efficient Omega-k
Range	IRW (m)	0.13125	0.13125	0.13163
	PSLR (dB)	−13.2643	−13.2658	−13.2535
	ISLR (dB)	−9.5756	−9.5762	−9.5749
Azimuth	IRW (°)	0.76875	0.76875	0.8500
	PSLR (dB)	−12.5289	−12.5355	−6.1867
	ISLR (dB)	−9.4189	−9.4248	−5.6380
